# Digital health and telemedicine in Pakistan: Improving maternal healthcare

**DOI:** 10.1016/j.amsu.2022.104425

**Published:** 2022-08-18

**Authors:** Wajeeha Bilal, Khulud Qamar, Amna Siddiqui, Prince Kumar, Mohammad Yasir Essar

**Affiliations:** Dow University of Health Sciences, Karachi, Pakistan; Karachi Medical and Dental College, Karachi, Pakistan; Dow University of Health Sciences, Karachi, Pakistan; Kabul University of Medical Sciences, Kabul, Afghanistan

**Keywords:** Telemedicine, Digital health, Maternal care, Pakistan

## Abstract

Pakistan has not benefited significantly from telemedicine, despite the promise that it could overcome many of the barriers impeding maternal healthcare delivery in emerging markets. Due to a lack of a regulatory framework and a lack of government interest, new companies in Pakistan have a hard time establishing healthcare projects that will be cost-effective and innovative. A review of telemedicine adoption in the past and present for improving maternal healthcare standards is presented in this article. Furthermore, a discussion of the challenges associated with digital health adoption is provided, as well as possible and feasible policies for making the use of digital health in maternal health more effective.

## 1 Introduction

Telemedicine is of great importance in low-income countries like Pakistan, where according to World Bank estimates 63% of the total population resides in rural areas, compared to only 37% in urban areas [1]. As compared to traditional care, telemedicine effectively caters to patients’ needs with greater convenience and lower cost, which is a combination preferred by most [2]. It makes healthcare accessible in remote and isolated places by cutting down transportation costs. Additionally, it saves a lot of commuting time, and patients do not have to take the day off from work, which is essential for many rural citizens due to their poor socio-economic conditions [3]. These factors make it more likely for the otherwise hesitant rural population to seek timely medical care. Telemedicine is essential in urban areas as well; it could ease the burden on understaffed healthcare facilities and prevent over-crowding as many patients receive medical care from the comfort of their homes [4].

In Pakistan, people from most rural areas must travel long distances to reach hospitals in cities which is expensive and dangerous to maternal and fetal health. Digital health services address these compulsions by connecting patients to their respective doctors through online means. It is reported from a few studies that telemedicine helped to save many lives of patients from Lower- and Middle-Income Countries (LMICs) due to complications that normally led to their deaths due to treatment being delayed [5]. Furthermore, telemedicine is a means to improve maternal care as it achieves more patient satisfaction because of more educational and supportive care with flexible meeting schedules [6]. Maternity care involves many events of emergency that require immediate interventions in presence of a health care provider (HCP). Telemedicine has been successful in monitoring maternal and fetal health during pregnancy, delivery, and post-birth when the patient is far from hospital settings [6]. From minor pregnancy complications such as vomiting, sweating, and mood swings to major emergencies like water breaking and preeclampsia, telemedicine has provided the most effective and patient-friendly interventions [6]. Telemedicine provides more feasible and cost-effective medical care by monitoring patient vitals with the use of mobile devices and smartphones, and interactions with physicians are carried through audio and video calls [2]. In a study consisting of 1060 maternal and fetal HCPs, 60% used telemedicine and responded enthusiastically to its use in the future [6].

Telehealth could also help provide reproductive counseling to women and girls in Pakistan. Recently, a joint effort by an NGO called Sehat Kahani Pakistan and Ipas (Intelligent Project Automation System) Pakistan has provided 3 days of online training to physicians, teaching them about digital health and telehealth consultations [7]. These physicians are now capable of providing virtual assistance to women for medical abortions and post-abortion contraception [7]. Also, 78 lady health care workers have been taught how to use telehealth services who in turn are reaching out to other girls and women and helping them access these free services [7].

As compared to traditional medical care, telemedicine regulates patient health even in critical settings and provides urgent treatment as seen with mobile stroke units in most high-income countries [2]. While such advanced and efficient mobile device facilities are yet to be introduced in Pakistan, telemedicine can be practiced simply through smartphones and internet connection.

Pakistan's reliance on telemedicine accelerated during the COVID-19 pandemic [8]. In March 2020, when the number of COVID cases was rising exponentially, the government was forced to impose a nationwide lockdown [8]. This along with the fear of getting infected with the virus resulted in many people opting to seek medical care from the comfort of their homes [8]. A study conducted at a tertiary care hospital in Pakistan found great success in classifying COVID patients according to the severity of their disease [9]. Furthermore, telemedicine facilities were set up by Faisalabad Medical University. Skye IDs (for video consultations) and WhatsApp numbers (for instant messaging) of physicians of different specialties were advertised in local print and electronic media [10]. These cyber consultations helped cater to more patients as it ensured patients with minor illnesses' stayed out of the hospital, and hence reduced the burden on overcrowded healthcare facilities [11]. Moreover, it also decreased the transmission of the virus and reduced the number of physicians at risk from close contact with COVID infected patients [8].

## 2 Challenges

In 2016, the World Health Organization (WHO) conducted a telemedicine survey and found that Pakistan didn't have any laws or regulations regarding the use of telemedicine [12]. Since health care has been devolved to the provinces, most provincial health departments are unaware of the benefits or existence of telemedicine. Without any regulations or framework, setting up any telemedicine program can be challenging. This is particularly true for international organizations that like a legal safety net before entering new markets [12]. Despite the contributions of various technologies, Pakistan's telemedicine operations remain at a primary level. Except for those regions affected by the earthquake, certain parts of the country lack telemedicine services [3]. Rural and remote regions face a variety of challenges that limit telehealth adoption and progress, despite technological advances. There are several challenges encountered by patients, including access to technology, acceptance of technology, relationship with the provider, and level of health literacy [13]. There is a lot of complexity in healthcare, and patients may find diagnoses difficult to comprehend. In the world of medicine, doctors face many challenges, including practicing “No-Touch” medicine, managing time, and building team relationships. Many of them also face issues with technology literacy, particularly those who were trained many years ago. Professional well-being as well as maintaining medical knowledge and coping with progressively increasing medical information are key challenges for providers [13]. Policy development and establishing a monitoring and adherence system will be the biggest challenges for the healthcare system of Pakistan. Physician accreditation and licensure, reimbursement systems, and liability may need to be reformed to provide reliable services. In addition, an information technology network, and well-trained staff will be required to ensure long-term success [13].

## 3 Efforts and recommendations

Obstetric-related-remote monitoring devices and other telemedicine services aim to try and address the maternal health and mortality crises. The former includes i.e. blood pressure monitors, blood glucose testing, and home-based fetal monitors. It not only monitors a patient's health and aids in reducing multiple antenatal and postnatal visits but can also be used to gauge whether a patient has breached the high-risk threshold and determine the need for immediate medical care [14]. Traditionally in Pakistan and other LMICs, many pregnant women suffer grave complications that lead to their deaths, these consequences are easily preventable if women are diagnosed and treated earlier. Postpartum Hemorrhage is one of the most common complications resulting in maternal mortality which occurs due to many reasons, including late administration of blood loss and lack of available blood products for transfusion [15]. A study in Latin America showed that a LMIC compared the implementation of digital health from traditional care on patients in Gynae and Obstetric wards and found a reduction of needing blood transfusion by 7%, Eclampsia by 5.5%, and a significant reduction in perinatal mortality by 29% [5]. They mentioned that regular monitoring of patients' health allowed efficient management which prevented any prognosis. According to severity, HCPs can direct the patient for the most efficient management. Mahdi S et al. emphasizes the introduction of digital health apps and portable sensors to monitor blood pressure, blood glucose, hormone levels, oxygen levels, and auscultation of viscera through glucometers, mobile blood pressure controls, pulse oximeters, and optical stethoscopes respectively [3]. This would provide regular monitoring of maternal and fetal health, indicating the slightest imbalance in body health immediately and preventing life-threatening consequences. An exploratory study by Sulaman H. et al., provides a favorable prospect for the uptake of telemedicine by obstetrics patients across Pakistan, following COVID-19 [16]. Further, the study of Akber S. et al. analyzed and reported the effectiveness of a mHealth intervention on infant and young child feeding among children ≤24 months of age in rural Islamabad over a six month duration [17]. Additionally, when the already practiced telemedicine in maternal care of the state is coupled with improving users’ experience (by training consultants and staff) and setting up effective communication in local languages can nurture the future use of telemedicine in obstetrics of the state (Table 1 highlights the adoption of telemedicine in maternal care.).

**Table 1:** Studies (meta-analyses) highlighting the adoption of telemedicine to manage and improve maternal care.

Study	Number of studies included in the synthesis	Study Characteristics	Patient Characteristics	Telemedicine Intervention(s)	Result(s)
Rahman MO et al., 2022 [18]	A total of 9 studies (10 articles) that randomized 10,348 pregnant women were included in this synthesis.	It considered Randomised controlled trials (RCTs).	The study included healthy pregnant women aged 15–49 years.	The tele-mediums considered to deliver antenatal care (ANC) such as through i.e. SMS text and voice messaging, voice calls, mobile vouchers, and animation film clips with the direction of i.e. 1-way communication or 2-way communications pertained to functions such as appointment reminders, health education or advice.	The study analyzed that ANC and skilled delivery care utilisation (SDCU) i.e. skilled birth attendance (SBA) during labour and facility delivery through mHealth can reduce maternal mortality.
Hanach N et al., 2021^19^	The meta-analysis included 20 randomized controlled trials with a total of 3252 patients.		The research subjects included, women who were 0 to 12-months postpartum, aged 18 years old or more, with a healthy pregnancy, and full-term birth. There was no restriction on the participant's baseline postnatal depression score.	The telemedicine-interventions comprised either telephone (e.g. telephone calls, text messages) or internet-based therapy (e.g. online psychoeducational sessions or CBT, mobile application, emails, video conference, social media platforms, and online chat).	Provides a guideline on effective mental health management of postpartum women suffering from i.e. postpartum depression (PPD) and anxiety via e-health.
Qian J et al., 2021^20^	A total of 15 RCTs with a total sample size of 4366 participates were included in the review.		The study population included pregnant or postpartum women. The age of the subjects ranged from 16 to 49 years, and the follow-up duration ranged from 24 hours to 6 months.	The study involved mHealth interventions, such as phone calls, text messages, and interactive computer systems, and the control group received usual care.	Interventions based on mHealth can significantly improve the rate of postpartum exclusive breastfeeding, breastfeeding efficacy, and participants' attitudes toward breastfeeding, and reduce health problems in infants.

To put Telemedicine in Pakistan into practice, regular audio and video calls are required with the respective doctor, using SMS, automated calls, applications, and telemedicine technologies. This necessitates that proper infrastructure with internet service and availability of mobile devices is to be provided in rural areas. Well-trained professionals, HCPs, and tech experts must be available to fix any difficulty the patients or attendants have while accessing digital health. Upon necessity, the HCP can also have the local health care centers connect with city hospitals on updates on maternal health [3]. In Pakistan, carrying out patient care through digital means requires the necessary infrastructure and funding in hospitals and rural areas. Additionally, the HCP must be qualified to utilize the technology suitable for themselves and the patient. HCPs must also educate the patient on using and connecting through digital health and enforce regularity in their usage [3]. Lastly, yet most important, telemedicine can allow long periods of communication between HCP and patients to ensure the understanding of procedures and diagnosis done by HCP. Patients can have their confusions and misconceptions answered through single calls/texts through telemedicine apps, as a study by Hasson S. et al. describes that communication is a safe and effective method to improve patient-physician relationship. Not only will this practice of providing medical knowledge result in an efficient care which gains trust of patients but also improves literacy towards telemedicine [18]. While initially implementing digital health with certain policies may be challenging, it proves to be compulsory to maintain the physical health of the community such as in times of COVID-19 and other unseen circumstances in the future.

## 4 Conclusion

There is no doubt that telehealth is gaining traction in the field of obstetrics. Various telehealth platforms enable obstetricians and gynecologists and other providers of obstetric and postpartum care to enhance prenatal, intrapartum, and postpartum care. Through maternal-fetal medicine telehealth, specialists and subspecialists can consult in a team-based fashion to improve quality and safety of obstetric practices in fragmented obstetric care delivery systems, especially in rural areas which have limited access to quality health care. Due to the high implementation costs, telehealth systems have not yet been adopted by low and middle-income nations. It is therefore essential to introduce more and better eHealth programs across the country through a constructive cycle of policy, execution, and evaluation.

## Ethical approval

N/A.

## Sources of funding

None.

## Author contribution

Wajeeha Bilal conceived the idea, came up with the design, wrote the abstract, challenges, conclusion, and edited the revised draft and organized references; Prince Kumar and Khulud Qamar wrote the introduction; Amna Siddiqui and Khulud Qamar wrote the efforts and recommendations; Mohammad Yasir Essar made the critical comments and revision. All authors revised and approved the final draft.

## Registration of research studies

1. Name of the registry: N/A
2. Unique Identifying number or registration ID: N/A
3. Hyperlink to your specific registration (must be publicly accessible and will be checked): N/A

## Guarantor

N/A.

## Consent

N/A.

## Provenance and peer review

Not commissioned, externally peer reviewed.

## Declaration of competing interest

None declared.
